# Copper(II)-Based Nano-Regulator Correlates Cuproptosis Burst and Sequential Immunogenic Cell Death for Synergistic Cancer Immunotherapy

**DOI:** 10.34133/bmr.0039

**Published:** 2024-06-27

**Authors:** Yingli Luo, Xianyu Luo, Yi Ru, Xinru Zhou, Didi Liu, Qian Huang, Maoyuan Linghu, Yuhang Wu, Zicheng Lv, Meimei Chen, Yinchu Ma, Yi Huang, Jilong Wang

**Affiliations:** ^1^ Wuxi School of Medicine, Jiangnan University, Wuxi, Jiangsu 214122, PR China.; ^2^ Affiliated Hospital of Jiangnan University, Jiangnan University, Wuxi, Jiangsu 214062, PR China.; ^3^ Joint Centre of Translational Medicine, Wenzhou Institute, University of Chinese Academy of Sciences, Wenzhou, Zhejiang 325000, PR China.; ^4^ Institute of Health and Medicine, Hefei Comprehensive National Science Center, Hefei 230601, China.

## Abstract

Immunogenic cell death (ICD) of tumor cells serves as a crucial initial signal in the activation of anti-tumor immune responses, holding marked promise in the field of tumor immunotherapy. However, low immunogenicity tumors pose challenges in achieving complete induction of ICD, thereby limiting the response rates of immunotherapy in clinical patients. The emergence of cuproptosis as a new form of regulated cell death has presented a promising strategy for enhanced immunotherapy of low immunogenic tumors. To trigger cuproptosis, copper-ionophore elesclomol (ES) had to be employed for the copper-transporting-mediated process. Herein, we proposed a copper(II)-based metal-organic framework nanoplatform (Cu-MOF) to facilitate a cooperative delivery of encapsulated ES and copper (ES-Cu-MOF) to induce cuproptosis burst and enhance ICD of fibrosarcoma. Our results showed that the ES-Cu-MOF nano-regulator could effectively release Cu^2+^ and ES in response to the intracellular environment, resulting in elevated mitochondrial ROS generation and initiated cuproptosis of tumor cells. Furthermore, sequential ICDs were significantly triggered via the ES-Cu-MOF nano-regulator to activate the anti-tumor immune response. The results of tumor inhibition experiment indicated that the nano-regulator of ES-Cu-MOF obviously accumulated in the tumor site, inducing ICD for dendritic cell activation. This enabled an increased infiltration of cytotoxic CD8^+^ T cells and consequently enhanced antitumor immune responses for successfully suppressing fibrosarcoma growth. Thus, the copper(II)-based metal-organic framework nano-regulator offered a promising approach for inducing cuproptosis and cuproptosis-stimulated ICD for cancer immunotherapy.

## Introduction

Cancer immunotherapy has been found to be highly effective in treating a variety of solid tumors, such as melanoma, lung cancer, and fibrosarcoma, by utilizing the immune system to recognize and eliminate cancerous cells [1–3]. This was achieved through a process known as immunogenic cancer cell death (ICD), which involves the release of antigens from dying tumor cells and the subsequent infiltration of cytotoxic T lymphocytes (CTLs) into the tumor site [4,5]. ICD could be induced by several modalities such as chemotherapies, radiotherapy, and photodynamic therapeutics, resulting in intracellular stress, including the generation of reactive oxygen species (ROS) and structural/functional changes of the endoplasmic reticulum (ER), as well as the release of damage-associated molecular patterns (DAMPs) [6–8]. These DAMPs, including high-mobility group box 1 protein (HMGB1), calreticulin (CARL), and adenosine triphosphate (ATP), would trigger the maturation and migration of DCs to enhance antitumor immunity [4,9–12]. Unfortunately, many tumors in the clinic show limited immunogenicity [13], making them difficult to be identified by the immune cells, thus allowing them to escape immune surveillance [14,15].

Recent studies have revealed that activating regulated cell death (RCD) including apoptosis, ferroptosis, pyroptosis, and necroptosis of tumor cells has the potential to stimulate antitumor immunogenicity and increase the efficiency of antitumor immunity therapies [16–18]. Ferroptosis was identified by the iron-dependent accumulation of lipid ROS to lethal levels, releasing HMGB1 in an autophagy-dependent manner [19–21]. For example, RSL-3, a ferroptosis inducer, has been shown to increase the immunogenicity of tumor cells, resulting in an increased infiltration of interferon (IFN)-γ^+^ T lymphocytes within the tumor and consequently improving immunotherapy [22,23]. Pyroptosis was a type of inflammatory cell death typically accompanied by activation of inflammasomes and maturation of pro-inflammatory cytokines interleukin-1β (IL-1β) and interleukin-18 (IL-18) [24]. Pyroptosis of tumor cells triggered the influx of anti-cancer immune cells, e.g., CD8^+^ T cells and NK cells, due to the release of DAMPs and cytokines [25,26]. Necroptosis was initiated by the activation of receptor-interacting protein kinases, leading to the release of immunogenic DAMPs [27]. Studies have revealed that necroptosis of tumor cells was capable of initiating the activation of the immune system, particularly the presentation of antigens and the cross-priming of CD8^+^ T cells, a process confirmed as ICD [28,29]. Utilizing drugs to trigger apoptosis and other forms of cell death in cancers has been employed as an effective means to alleviate immunosuppression and stimulate systemic immune responses against tumors [16]. The discovery of new types of RCD and their role in immunity and tumor formation has reinvigorated the development of anti-cancer treatments.

Cuproptosis was a novel type of RCD characterized by an excessive amount of copper intracellularly [30]. Research has revealed that cuproptosis was a singular copper-dependent death pathway, which leads to the clustering of mitochondrial lipoylated proteins and the destabilization of FDX1 (a reductase known to reduce Cu^2+^ to its more toxic form, Cu^1+^, and to be a direct target of elesclomol [ES]), in the tricarboxylic acid (TCA) cycle. Meanwhile, DLAT (dihydrolipoamide *S*-acetyltransferase), a component of the pyruvate dehydrogenase (PDH) complex, plays a critical role in the process of cuproptosis. The mechanism of DLAT in cuproptosis involves copper binding directly to lipoylated components of the TCA cycle, specifically affecting the PDH complex [31]. The process of cuproptosis was significantly reliant on ionophores such as ES to facilitate the transfer of extracellular copper to cellular mitochondria in an FDX1-dependent manner [32]. Cuproptosis has been shown to have significant therapeutic implications in cancer and would potentially be exploited in cancer immunotherapy [30,33]. Several studies have reported the development of cuproptosis for cancer therapy. For example, Xu et al. reported the synthesis of a photothermally activated nanoplatform that contained the cuproptosis inducer, disulfiram (DSF), as a potential synergistic therapy for breast cancer. The DSF-loaded copper-doped Au@MSN nanoplatform exhibited high stability and maximized its therapeutic effect while minimizing adverse effects on healthy cells [34]. Guo et al. [35] unveiled the synthesis of encapsulated ES and Cu nanoparticles that were responsive to ROS, combined with αPD-L1, which enhanced the synergistic effects of combining copper-based cuproptosis induction and immune checkpoint blockade. It has been verified that intracellular copper accumulation could induce mitochondrial stress and ultimately lead to cuproptosis, while simultaneously amplifying ROS generation and causing oxidative damage to the cellular components [36,37]. Thus, cuproptosis has become a potential target to boost antitumor immunogenicity and improve antitumor treatments.

**Fig. 1.**
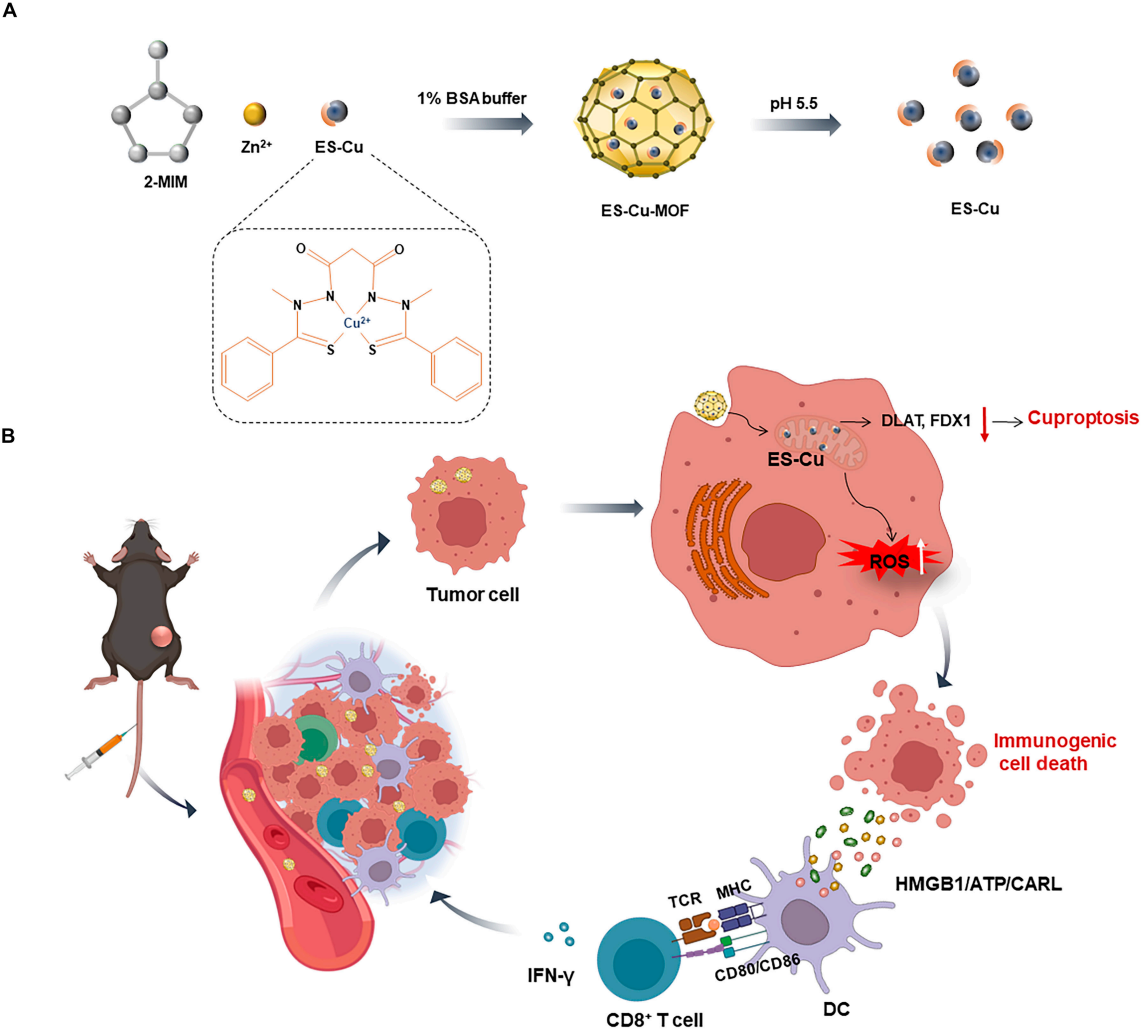
(A) A schematic illustration of the preparation of a copper(II)-based metal-organic framework nano-regulator (ES-Cu-MOF). (B) An ES-Cu-MOF nano-regulator could be utilized to mediate cuproptosis and initiate ICD to suppress tumor growth and enhance antitumor immune responses. The ES-Cu-MOF nano-regulator effectively delivered Cu^2+^ and ES to tumor cells based on the intracellular environment. As a result, ES chelated Cu^2+^ to enhance its accumulation in mitochondria. The accumulation of copper and ES led to the depletion of DLAT and FDX1, disrupting mitochondrial function and inducing the production of reactive oxygen species (ROS), thus initiating the cuproptosis process. Furthermore, the ES-Cu-MOF-mediated cuproptosis enhanced the immunogenicity of tumor cells by causing the exposure or release of damage-associated molecular patterns (DAMPs), such as ATP, calreticulin (CARL), or HMGB1. The presence of DAMPs could activate dendritic cells (DCs) and enhance the infiltration of cytotoxic CD8^+^ T cell, stimulating a robust anti-tumor immune response. Some graphical elements created with biorender.com.

Research on metal-organic frameworks (MOFs) has been gaining traction as a novel nanocarrier for drug delivery in recent years due to their unique properties for targeted drug delivery [38–41]. One of the most striking features of MOFs was their exceptionally high specific surface area, which facilitates a substantial drug loading capacity. This attribute is crucial for achieving efficient and sustained drug release profiles, ensuring that therapeutic agents were delivered at optimal concentrations over extended periods. Furthermore, the customizable pore size and functionalization of MOF surfaces also contributed to the targeted delivery of drugs, enhancing the selectivity and efficacy of treatments by directing therapeutic agents specifically to diseased tissues or cells. Another noteworthy advantage of MOFs in drug delivery was their high degree of structural tunability. The ability to modify the composition and functionality of MOFs at the molecular level allowed for the optimization of drug release kinetics, ensuring that drugs are released in a controlled manner that aligns with the therapeutic needs of patients. Their potential to significantly improve the efficacy and safety of therapeutic interventions has made MOFs a focal point of current research and development efforts in the field of nanomedicine. The porous materials, composed of metal ions (e.g., Fe, Cu, and Mn) connected to organic ligands, spontaneously release metal ions upon internalization into tumor cells [42]. In this regard, copper(II)-based MOFs (Cu-MOFs) have gained significant attention for the delivery of anti-cancer drug copper ionophores ES. Herein, a Cu-MOF nanoplatform was designed to incorporate cuproptosis-based agents ES, thus synergistically creating an efficient and targeted nanoplatform for cancer immunotherapy. The integration of ES into a Cu-MOF (ES-Cu-MOF) nano-regulator was capable of delivering targeted substances to tumor cells, releasing Cu^2+^ and ES in response to the intracellular environment and triggering cuproptosis and immunogenic cell death (ICD) of fibrosarcoma simultaneously. ES-Cu-MOF nanoparticles could lead to the depletion of the FDX1 and an augmented production of mitochondrial ROS, both of which were consequences of the cuproptosis of tumor cells. Furthermore, the ES-Cu-MOF nanoparticles were also able to trigger ICD of tumor cells to activate the immune system in vivo and in vitro. Intravenous injection of the ES-Cu-MOF nanoparticles could accumulate in the tumor site and inhibit the growth of fibrosarcoma by triggering cuproptosis of tumor cells and ICD for DC activation. The nano-regulator not only increased cytotoxic CD8^+^ T infiltration, but also established systemic immune memory, which significantly improved antitumor immune responses (Fig. 1). The incorporation of ES into a Cu-MOF nanoplatform provides a unique opportunity to exploit the therapeutic potential of copper-dependent RCD and stimulate ICD for antitumor immunity activation while minimizing adverse effects.

## Materials and Methods

### Materials and reagents

Zinc nitrate hexahydrate (Zn(NO_3_)_2_·6H_2_O) (Catalog: 10196-18-6) and Copper(II) nitrate trihydrate (Cu(NO_3_)_2_·3H_2_O) (Catalog: 10007918) were purchased from Sinopharm. Dimethyl imidazole (2-MIM) was obtained from Shanghai Titan (Catalog: 1106319). ES (Catalog: 48832-69-5) and Cell Counting Kit-8 (CCK8) (Catalog: c0005) were purchased from TargetMol. Bovine serum albumin (BSA) was purchased from Biosharp (Catalog: BS114-100g). MitoSOX was obtained from Invitrogen. Cy5 and H_2_DCFDA were purchased from Thermo Fisher Scientific. Anti-mouse FDX antibody (T510671), anti-mouse DLAT antibody (T58125), anti-mouse HSP70 antibody (M20033), anti-mouse HMGB1 (PHX1604S), anti-mouse CALR (PHJ3867S) and anti-mouse β-actin (P30002) were purchased from Abmart. Alexa Fluor 568 donkey anti-rabbit IgG H&L antibody (ab175470) was purchased from Abcam. The ATP assay kit (S0027) was purchased from Beyotime. Anti-mouse CD16/32 (clone: 93), BV510 anti-mouse CD45 (clone: 30-F11), BV421 anti-mouse CD3 (clone: 17A2), PE-Cy7 anti-mouse CD4 (clone: RM4-5), BV650 anti-mouse CD8 (clone: 53-6.7), FITC anti-mouse CD44 (clone: IM7), PE anti-mouse CD62L (clone: MEL-14), PE/Cy7 anti-mouse IFN-γ (clone: XMG1.2), PerCP-Cy5.5 anti-mouse CD11c (clone: N418), BV605 anti-mouse I-A/I-E (clone: M5/114.15.2), Alexa Fluor 488 anti-mouse CD80 (clone: 16-10A1), and APC/Cy7 anti-mouse CD86 (clone: GL-1) were purchased from BioLegend. Granulocyte-macrophage colony-stimulating factor (GM-CSF) (CK02) and interline-4 (CK74) were purchased from Novoprotein. Dulbecco minimum essential medium (DMEM) (MA0212-2) and RIMP 1640 medium (MA0215-2) were purchased from Meilunbio. Fetal bovine serum (FBS) was purchased from EallBio (03.C16001DC). Culture plates were purchased from NEST biotechnology (Wuxi, China). The solvents and materials were utilized in strict adherence to the provided guidelines, without any additional alterations.

### Cell culture and mice

The American Type Culture Collection was the source of the MCA205 mouse fibrosarcoma cells and HT1080 human fibrosarcoma cells, which were cultured in DMEM supplemented with 10% v/v FBS and 1% penicillin/streptomycin. The incubation was conducted in plates, at a temperature of 37 °C and a CO_2_ concentration of 5%. Five- to 8-week-old male C57BL/6 mice were purchased from GemPharmatech Co. Ltd. All mice were bred in a controlled environment at Jiangnan University’s animal facility, where the temperature was maintained at 22 ± 2 °C and humidity at 50 ± 15%, with a 12-h dark–light cycle.

The Animal Care and Use Committee at Jiangnan University (JN.No20220930c0600228[387]) approved all animal experiments conducted in accordance with the guidelines outlined in the Guide for the Care and Use of Laboratory Animals of Jiangnan University.

### Isolation and culture of bone marrow-derived dendritic cells

Female C57BL/6 mice were used to isolate bone marrow-derived dendritic cells (BMDCs) from their hind limbs. The BMDCs were cultured in RPMI 1640 medium, which contained 10% heat-inactivated FBS, 1% penicillin/streptomycin, GM-CSF (20 ng/ml), and IL-4 (5 ng/ml). The cells were seeded into 24-well plates at a density of 1 × 10^6^ cells/ml and cultured in a CO_2_ incubator at 37 °C with 5% CO_2_. On day 3, the culture medium was replaced with fresh medium supplemented with GM-CSF and IL-4 to remove any unattached cells and cell debris. Semi-suspended cells and loosely adherent cells were collected for further study between days 7 and 9.

### Synthesis and characterization of Cu-MOF and ES-Cu-MOF

Cu-MOF nanoparticles were prepared in a methanol system. Utilizing a succinct approach, solution A was formed by blending 5.5 mg of Zn(NO_3_)_2_·6H_2_O and 3 mg of Cu(NO_3_)_2_·3H_2_O in 2.5 ml of methanol. Separately, 2.5 mg of methanol was employed to dissolve 125 mg of 2-MIM, forming solution B. The resulting solution A was added gradually to solution B with rigorous mixing over a time frame of 1 h. Then, the Cu-MOF nanoparticles were extracted through centrifugation (at 4 °C, 8,000 rpm, for 10 min) and subjected to a triple methanol washing regimen, resulting in the acquisition of the Cu-MOF nanoparticles. Incorporating ES into the reaction required a 100 mM solution of 50 μl of ES to be mixed with Solution B with sonication for 3 min at 80 W. This was done using a Vibra-Cell VCX130 (Sonics & Materials, Inc., Newtown, USA) over an ice bath. The subsequent mixture was then processed according to the established procedure to acquire the ES-Cu-MOF nanoparticles. Afterwards, the nanoparticles were reconstituted in a solution containing 1% BSA in ultrapure water or 1×PBS buffer for subsequent experimentation. To determine the diameters and zeta potentials of the nanoparticles, a dynamic light scattering (DLS) technique was utilized with the Malvern Zetasizer Nano ZS90 (Worcestershire, UK). The morphology of the nanoparticles was examined at a concentration of 1 mg/ml by utilizing the transmission electron microscope JEM-2100Plus (JEOL, Akijima, Japan). The encapsulation efficiency of ES was gauged through HPLC using a C18 column with a UV absorption wavelength of 254 nm and the encapsulation efficiency of Cu was assessed by a copper probe (R6G, heliosense). Cu-MOF and ES-Cu-MOF were also measured by XRD patterns and XPS spectra. The existence of C, N, O, Cu, and Zn in the Cu/ZIF-8 structure was also evidenced via x-ray photoelectron spectroscopy (XPS) analysis. Absorption properties of Cu-MOF and ES-Cu-MOF were measured with N_2_ adsorption–desorption analysis.

### The pH-activated release of Cu^2+^ and ES in vitro

To analyze the degradation of the ES-Cu-MOF nanoparticles, a solution was formulated by blending 1 mg of the particles in 2 ml of the relevant release buffer, either at pH 5.5 or 7.5, and maintained at a temperature of 37 °C. The resulting supernatant was collected at different time points after agitation and filtration through a dialysis bag with a molecular weight cutoff of 14,000 Dalton. Following the process, the supernatant underwent lyophilization. The concentration of Cu^2+^ released into the supernatant was measured using a copper probe (R6G, heliosense). The concentration of ES released from the nanoparticles was measured using HPLC using C18 column with the UV absorption wavelength of 254 nm.

### Cellular uptake of Cu-MOF nanoparticles in vitro

To investigate the cellular uptake of Cu-MOF nanoparticles, Ce6-loaded Cu-MOF nanoparticles were synthesized and employed. MCA205 cells were cultured in 24-well plates for 24 h prior to being subjected to Cu-MOF_Ce6_ for 1, 3, or 6 h at 37 °C. The cells were then washed with 1×PBS buffer 3 times and collected for flow cytometry analysis using Accuri C6 Plus by BD in the USA. Additionally, MCA205 cells were cultured in 24-well plates for 24 h and then treated with Cu-MOF _Ce6_ for 1, 3, or 6 h. Subsequently, the cells that had undergone treatment were subjected to a gentle wash with fresh PBS. After this, they were fixed with 4% paraformaldehyde for a duration of 15 min and permeabilized with Triton X-100 in PBS (0.3%) for 15 min. Following this, the fixed cells were stained with DAPI for a duration of 5 min. The uptake of cells in MCA205 cells was visualized using CLSM.

### Cell viability assays

To evaluate the cytotoxicity of the Cu-MOF nanoparticles, MCA205 cells were seeded in 96-well plates at a density of 1×10^4^ cells/well and allowed to attach for 24 h. Subsequently, the cells were exposed to varying concentrations of PBS, ES, Cu-MOF or ES-Cu-MOF ([ES] = 0, 2.5, 5, and 10 μM) and incubated for 6 h at 37 °C. Thereafter, 10 μl of CCK8 solution was added and the cells were allowed to incubate for an additional hour. To assess the viability of the cells, a BioTek Epoch was employed to collect the CCK8-containing medium, and the assay was conducted at a wavelength of 490 nm. To assess cell cytotoxicity at different time points, MCA205 cells were seeded in 96-well plates at a density of 1 × 10^4^ cells/well, cultured overnight, and then exposed to PBS, ES, Cu-MOF, or ES-Cu-MOF ([ES] = 5 μM) for 1, 3, 6, or 12 h. All treated cells were then analyzed using CCK8, as per the aforementioned method.

### Detection of mitochondrial and intracellular ROS generation

The measurement of mitochondrial and intracellular ROS generation involved the seeding of MCA205 cells in a 24-well plate at a density of 1 × 10^5^ cells/well, followed by overnight culture. The cells were then subjected to treatment with PBS, ES, Cu-MOF, or ES-Cu-MOF for a period of 6 h. After treatment, the cells were stained with MitoSOX (5 μM) or H_2_DCFDA probe and cultured for 60 min. The fluorescence microscope (Axio Vert A1, Carl Zeiss AG, Germany) was used to observe the mitochondrial and intracellular ROS generation in the treated cells.

### Western blotting analysis

To measure the cuproptosis-associated protein, 1×10^5^ cells/well of MCA205 cells were seeded in a 24-well plate and incubated overnight. The cells were subsequently incubated with various substances, including PBS, ES, Cu-MOF, ES-Cu-MOF, or ES-Cu, for a period of 6 h. Following treatment, all cells were extracted using NP-40 lysis buffer containing complete protease inhibitor. The amount of total protein was determined using the BCA assay and was equalized before loading. The proteins were then subjected to detection using anti-mouse FDX1 antibody, anti-mouse DLAT antibody (diluted 1:2,000 with 1% BSA), anti-mouse HSP70 antibody (diluted 1:2,000 with 1% BSA), and anti-mouse β-actin (diluted 1:5,000 with 1% BSA) through Western blotting, in accordance with standard protocols.

To measure the protein associated with ICD, 1 × 10^5^ cells/well of MCA205 cells were seeded in a 24-well plate and incubated overnight. The cells were then treated with various substances, including PBS, ES, Cu-MOF, ES-Cu-MOF, or ES-Cu, for a duration of 6 h. After treatment, the precipitated supernatants were analyzed using anti-mouse HMGB1 antibody (diluted 1:2,000 with 1% BSA) and anti-mouse CARL antibody (diluted 1:2,000 with 1% BSA) through Western blotting, following standard protocols.

### In vitro investigation of ICD

To gain further insight into the induction of ICD, an in vitro analysis was conducted to assess the surface expression of CARL. MCA205 cells were exposed to various treatments, including PBS, ES, Cu-MOF, ES-Cu-MOF, or ES-Cu, for a period of 6 h. The cells were collected and then exposed to an anti-mouse CARL antibody overnight at 4 °C, which was followed by a secondary antibody, Alexa Fluor 568 donkey anti-rabbit IgG, for an hour at room temperature. DAPI staining was conducted and the cells were then viewed using CLSM. Furthermore, the supernatant was collected post-treatment, and the extracellular ATP release was measured using an ATP assay kit (S0027).

### In vitro analysis of the activation of DCs

The MCA205 cells were seeded in a 24-well plate with a density of 1×10^5^ cells/well and cultured overnight. The cells were then treated with PBS, ES, Cu-MOF, ES-Cu-MOF, or ES-Cu ([ES] =2.5 μM) for 6 h. Afterward, immature BMDCs were added to the cells and cocultured for an additional 24 h. The harvested BMDCs were treated with anti-mouse CD16/32 to block nonspecific binding and then stained with PerCP-Cy5.5 anti-mouse CD11c, BV605 anti-mouse I-A/I-E, Alexa Fluor 488 anti-mouse CD80, and APC/Cy7 anti-mouse CD86 for 30 min at 4 °C. The maturation status of the DCs was analyzed using a BD Celesta flow cytometer.

### In vitro analysis of the proliferation of T cells

Initially, BMDCs were subjected to the aforementioned treatment. Subsequently, they were cocultured with MCA205 cells that had been treated with PBS, ES, Cu-MOF, ES-Cu-MOF, or ES-Cu for 24 h. Isolated CD8^+^ T cells from the spleens of C57BL/6 mice using the MojoSort Mouse CD8 T Cell Isolation Kit (BioLegend, #423801) were labeled with a CFSE Cell Division Tracker Kit (BioLegend, #480035) and subsequently added to the culture. After 48 h of coculture, the proliferation of the CFSE-labeled CD8^+^ T cells was analyzed using flow cytometry.

### Tumor model establishment

To assess the therapeutic effects in vivo, a MCA205 subcutaneous xenograft model was established. Mice (aged 8 weeks) were inoculated with 5×10^5^ MCA205 tumor cells subcutaneously and the tumor-bearing mice were randomly assigned to treatment groups. When the tumor volume had reached approximately 50 mm^3^, the mice were utilized for in vivo study.

#### In vivo biodistribution study

The mice-bearing tumors were administered an intravenous injection of Cu-MOF_Ce6_ ([Ce6] =2 mg/kg), which was labeled with Ce6. Following the administration, biodistribution images were collected at 6 different time intervals (1 h, 3 h, 8 h, 12 h, 24 h, and 48 h) using the In Vivo Imaging System (IVIS SPECTRUM, PerkinElmer, Ex/Em = 650 nm/670 nm). The ex vivo biodistribution study was conducted by sacrificing the mice after 48 h from the injection and collecting the ex vivo imaging of organs, including the liver, spleen, lung, kidney, and tumor, followed by quantitative analyses using the IVIS Spectrum imaging system.

### In vivo anti-tumor therapy

When the MCA205 tumors reached a size of approximately 50 mm^3^, a group of mice were randomly divided into 4 groups consisting of 5 mice each. The groups were as follows: a control group that received PBS, a group that received ES, a group that received Cu-MOF, and a group that received ES-Cu-MOF. After 14 days, the mice were given intravenous injections every 2 days, with a dose of 30 mg ES per kg of body weight. For a period of 20 days, the tumor sizes and body weights of the mice were monitored every other day. The tumor volume was calculated using the formula: volume = (length × width^2^)/2. Following the treatment, the mice were humanely euthanized and the tumors were removed and weighed. The survival of each group was monitored and tracked on a daily basis.

### In vivo immune responses

After treatment, tumor-draining lymph nodes (TDLNs) and tumor tissues were excised. The TDLNs were placed in a dish and separated into single cells by pressing with the plunger. The tumor tissues were cut into small pieces and digested in RPMI 1640 medium with 1 mg/ml collagenase IV, and 0.1 mg/ml hyaluronidase at 37 °C for 1 h. Prior to surface staining, the harvested cells were treated with anti-mouse CD16/32 for 20 min on ice to block nonspecific binding. Subsequently, multiparameter staining was further conducted using fluorophore-labeled antibodies for 30 min at 4 °C including BV510 anti-mouse CD45, BV421 anti-mouse CD3, PE-Cy7 anti-mouse CD4, BV650 anti-mouse CD8, FITC anti-mouse CD44, PE anti-mouse CD62L, PerCP-Cy5.5 anti-mouse CD11c, BV605 anti-mouse I-A/I-E, Alexa Fluor 488 anti-mouse CD80, and APC/Cy7 anti-mouse CD86. Then, cells were stained with DAPI and analyzed using a BD flow cytometry Celesta flow cytometer. For the analysis of intracellular IFN-γ, the cells were treated with Cell activation Cocktail (with Brefeldin A) (BioLegend) and then fixed and permeabilized with a BD Cytofix/CytopermTM Fixation/Permeabilization Kit (BD Biosciences) before being stained with PE/Cy7 anti-mouse IFN-γ antibodies. Then, cells were analyzed using a BD flow cytometry Celesta flow cytometer. Flow cytometry data were analyzed with FlowJo version 10.

### In vivo systemic immune responses

To inspect the function of ES-Cu-MOF in eliciting the systemic immune responses of ICD, mice bearing both primary and distant tumors were established. PBS- or ES-Cu-MOF ([ES] = 2.5 μM for 3 h)-treated MCA205 cells (5 × 10^5^ cells each mouse) were injected subcutaneously in the right lower flank of mice as primary tumors. After 7 days, MCA205 cells (5 × 10^5^ cells each mouse) were injected subcutaneously in the left lower flank of mice to form distant tumors. The tumor sizes of the mice were monitored every other day for a period of 20 days. To accurately evaluate the infiltration of immune cells, both primary and distant tumors were collected on day 21 and then analyzed with flow cytometry according to the specified protocol.

### Statistical analysis

Statistical analysis was accomplished with GraphPad Prism 8.3.0 software, and ordinary one-way analysis of variance (ANOVA) and 2-tailed Student’s *t*-test were applied to determine significance. Results were considered statistically significant for *P < 0.05; **P < 0.01; ***P < 0.001; ****P < 0.0001; any other value was considered no significant difference, with all data expressed as means ± standard deviation.

## Results

### Preparation and characterization of ES-Cu-MOF nanoparticles

**Fig. 2.**
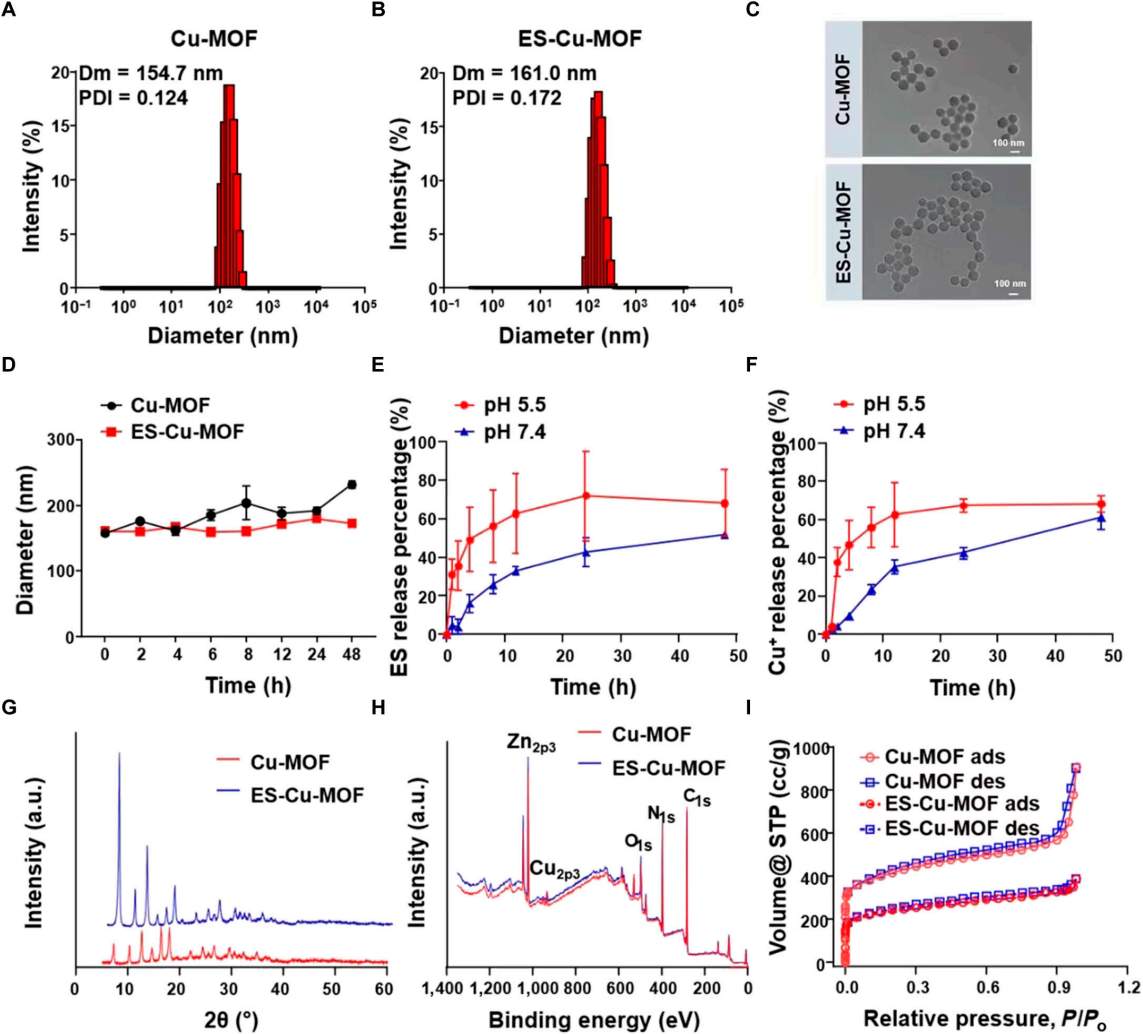
The characterization of Cu-MOF and ES-Cu-MOF nanoplatform. (A and B) Hydrodynamic diameter and zeta potential of Cu-MOF and ES-Cu-MOF. (C) TEM images of Cu-MOF and ES-Cu-MOF. (D) Size change curves of Cu-MOF and ES-Cu-MOF incubated with 1 × PBS over time. (E and F) Cumulative release profiles of ES and Cu^2+^ from ES-Cu-MOF at different pH conditions (7.4 and 5.5). (G) XRD patterns of Cu-MOF and ES-Cu-MOF. (H) XPS spectra of Cu-MOF and ES-Cu-MOF. (I) Absorption properties of Cu-MOF and ES-Cu-MOF were measured with N_2_ adsorption–desorption analysis. Data presented as mean ± SEM.

Cu-MOF nanoparticles were synthesized using Zn (NO_3_)_2_·6H_2_O, Cu (NO_3_)_2_·3H_2_O, and 2-MIM in a methanol system for the purpose of creating a nano-regulator for systemic and intracellular collaborative delivery of Cu^2+^ and ES. Additionally, ES was incorporated into the Cu-MOF nanoparticles. The size and morphology of the Cu-MOF and ES-Cu-MOF nanoparticles were characterized using DLS and transmission electron microscopy (TEM). Figure 2A and B reveal that both had a single-peak hydrodynamic size distribution and an average size of ~154.7 nm and ~161.0 nm, respectively. TEM images also showed that both had a spherical shape with an average size of ~150 nm (Fig. 2C). Additionally, the Cu-MOF and ES-Cu-MOF nanoparticles maintained their stability in PBS buffer over 48 h, with only a slight change in size (Fig. 2D). The ES drug loading capacity of the Cu-MOF nanoparticles was determined to be 58.05%, while the Cu^2+^ loading capacity of the Cu-MOF nanoparticles was calculated to be 63.42%. To assess the release profile of ES-Cu-MOF in extracellular and intracellular environments, the release content of ES and Cu^2+^ was measured after incubation of ES-Cu-MOF nanoparticles in PBS (pH 7.4 and pH 5.5) at 37 °C. The ES-Cu-MOF nanoparticles exhibited stability with a low release of ES at pH 7.4, with less than 30% released after 12 h of incubation. At pH 5.5, a linear ES release was witnessed after 6 h, reaching 62% within 12 h (Fig. 2E). The release of Cu^2+^ was also monitored using a copper probe, following a similar release profile to ES and reaching a total of 60% after 24 h at pH 5.5 (Fig. 2F). These findings indicated that ES-Cu-MOF nano-regulators were able to effectively and quickly discharge ES and Cu^2+^ in a simulated intracellular endosomal microenvironment (pH 5.5). As shown in Table S1, the S element was 16.616% in ES-Cu-MOF after ES loaded compared with 0% in Cu-MOF, which indicated that ES was successfully loaded into the ES-Cu-MOF. A set of sharp peaks of typical of well-crystallized ZIF-8 occurred in the XRD patterns of Cu-MOF and ES-Cu-MOF (Fig. 2G), which showed that ES-Cu-MOF could maintain a stable and integrate crystallized structure after ES loaded. As shown in Fig. 2H, the existence of C, N, O, Cu, and Zn in the Cu/ZIF-8 structure was also evidenced via XPS analysis. The absorption properties of Cu-MOF and ES-Cu-MOF were measured with N_2_ adsorption–desorption analysis. The surface area of BET was 1,326 m^2^/g and 770 m^2^/g for Cu-MOF and ES-Cu-MOF, respectively (Fig. 2I).

### ES-Cu-MOF nano-regulators enhance the cellular uptake and induce cuproptosis efficacy in vitro

**Fig. 3.**
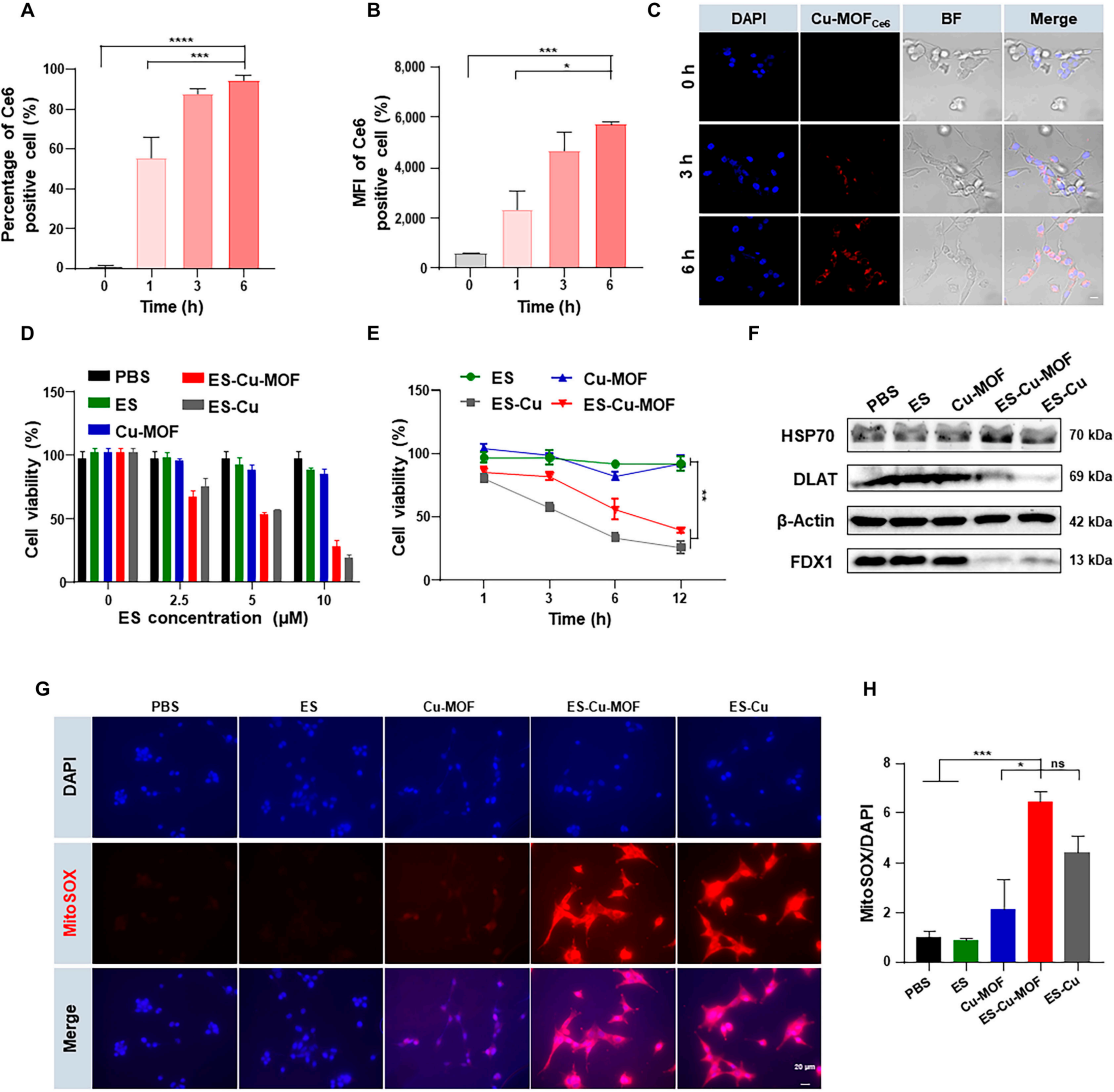
In vitro intracellular uptake and cuproptosis induction of the ES-Cu-MOF nano-regulator. (A and B) The percentage and mean fluorescence intensity (MFI) of Ce6-positive cells in MCA205 cells were measured by flow cytometry after treatment at different time points. (C) Immunofluorescence detection of cell internalization treatment with Cu-MOF_Ce6_ in MCA205 cells. (D) Cell viability of MCA205 cells treated with the indicated concentrations of elesclomol for 6 h. (E) Cell viability of MCA205 cells in various treatments at different time points. (F) The Western blot analysis of FDX1, DLA,T and HSP70 expression following 6-h pulse treatments of various types. (G) Confocal microscopy analysis in MCA205 cells treated with different formulations, followed by staining with MitoSOX and DAPI. (H) The relative fluorescence intensity of mitoSOX/DAPI in MCA205 cells after different treatments. Data presented as mean ± SEM. *P < 0.05; **P < 0.01; ***P < 0.001; ****P < 0.0001.

Subsequently, we proceeded to investigate the cellular uptake of ES-Cu-MOF as ES was key in copper-induced cuproptosis of tumor cells. The cellular uptake ability of ES-Cu-MOF was investigated using fluorescence Ce6 loaded nanoparticles, resulting in the formation of Cu-MOF_Ce6_. Following this, MCA205 cells were incubated with Cu-MOF_Ce6_ for various time periods. Flow cytometry analysis revealed that the percentage of Ce6 fluorescent positive cells increased from 1 h to 3 h, reaching up to 85%, and showed no significant difference between 3 and 6 h (Fig. 3A). Additionally, the fluorescence intensity of MCA205 cells after 3 and 6 h was observed to be approximately threefold that of 1 h (Fig. 3B). Furthermore, the intracellular fluorescence of Cu-MOF_Ce6_ was studied using confocal laser scanning microscopy (CLSM) analysis (Fig. 3C). The results were consistent with those derived from flow cytometry analysis, thus indicating that the Cu-MOF nanoparticles could effectively deliver the drug to MCA205 cells.

After incubation with ES-Cu-MOF nano-regulators, intracellular copper ion was detected using copper probe. To serve as a positive control, a mixture of ES and CuCl_2_ (ES-Cu) was selected, which could induce typical cuproptosis. The results showed that the intracellular copper content of ES-Cu-MOF was significantly higher than that of PBS and ES, indicating that the nanoplatforms could effectively increase intracellular copper concentration (Fig. S1A). Subsequently, the cytotoxicity of ES-Cu-MOF was determined using the CCK8 assay. As shown in Fig. 3D, the ES-Cu-MOF nano-regulators exhibited a dose-dependent cytotoxicity on MCA205 cells, the same as the ES-Cu group. This was significantly lower than that of free ES and Cu-MOF. The ES-Cu-MOF nano-regulators were observed to induce time-dependent cell death, displaying a maximum of 60.6% at 12 h, comparable to the positive control (Fig. 3E). Furthermore, the cytotoxicity on human fibrosarcoma HT1080 cells was shown to be dose-dependent (Fig. S1B). These results indicated that ES-Cu-MOF nano-regulators could effectively induce cell death in both MCA205 and HT1080 cells.

In order to validate that the cell death of ES-Cu-MOF nano-regulators was caused by cuproptosis, additional verification is required. The level of cuproptosis-related proteins was measured using Western blotting after treatment in MCA205 cells. The results showed that the expression of DLAT and FDX1 significantly reduced and levels of HSP70 were increased in the ES-Cu-MOF group compared to the PBS and ES (Fig. 3F and Fig. S1C and D). To further investigate this, FDX1-knockout HT1080 cells (HT1080-sgFDX1) were established and confirmed using Western blotting (Fig. S1E). Then, HT1080 cells and HT1080-sgFDX1 cells were treated with ES-Cu-MOF. The results showed that the cell death was significantly higher in HT1080 cells than in the HT1080-sgFDX1 cells (Fig. S1F), suggesting that FDX1-knockout partially rescued cells from ES-Cu-MOF induced cell death. Studies have reported that ES selectively transferred extracellular Cu(II) to mitochondria, where the accumulation of Cu(II) induces ROS production and triggers cuproptosis [33]. To further assess the capacity of ES-Cu-MOF to induce cuproptosis, the mitochondrial and intracellular ROS production of MCA205 cells was measured after incubation of nanoparticles for 6 h. It was observed that the mitochondrial ROS production of ES-Cu-MOF and ES-Cu groups was higher than that of PBS and Free ES groups (Fig. 3G and H). As evidenced in Fig. S1G, the intracellular total ROS production of ES-Cu-MOF and ES-Cu groups was also notably augmented. Therefore, these results indicated that ES-Cu-MOF nano-regulators were able to effectively induce cuproptosis in both MCA205 and HT1080 cells.

### ICD effect induced by ES-Cu-MOF nano-regulators in vitro

**Fig. 4.**
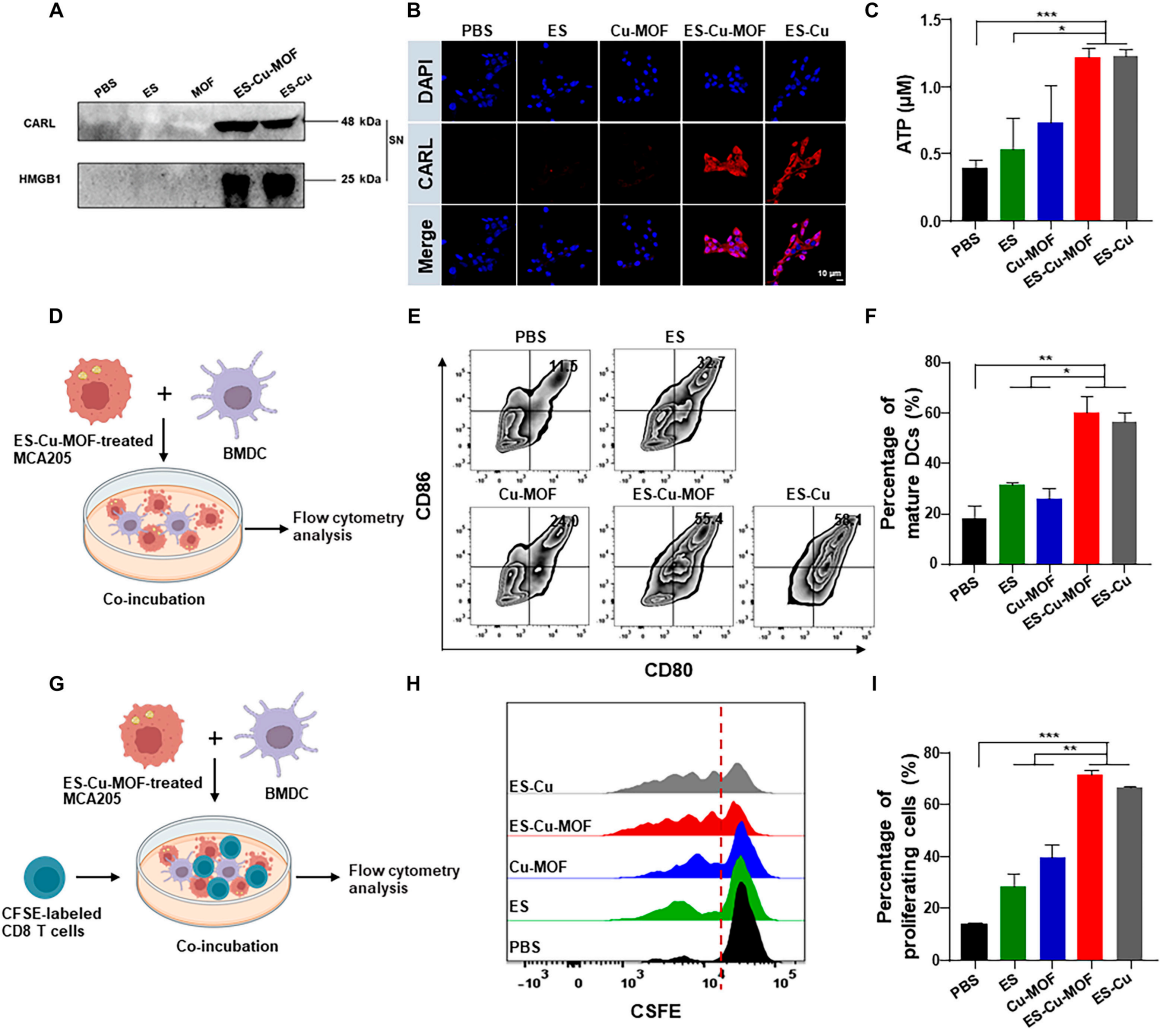
In vitro ICD induction and immune activation of the ES-Cu-MOF nano-regulator. (A) The expression of CARL and HMGB1 in the supernatant of MCA205 cells after different treatments by Western blot. (B) Immunofluorescence staining of CARL in MCA205 cells after different treatments by confocal microscopy; nuclei were stained by DAPI (blue) and CARL on cell surface were stained by anti-mouse CARL antibody and Alexa Fluor 568 donkey anti-rabbit IgG (red). (C) The release of ATP in the supernatant of MCA205 cells after different treatments by the ATP assay kit. (D) Schematic representation of experimental design for in vitro the MCA205 cells–BMDCs coculture assay. (E) Representative flow cytometric plots illustrating the expressions of CD80 and CD86 in BMDCs after coculture. (F) Statistical percentages of CD80^+^ and CD86^+^ mature BMDCs after coculture. (G) Schematic representation of experimental design for in vitro MCA205 cells–BMDCs–CFSE-labeled CD8^+^ T cells coculture assay. (H) Representative flow cytometry histograms illustrating the CFSE fluorescence intensities of CD8^+^ T cells following various treatments. (I) Statistical percentages of proliferative CD8^+^ T cells after coculture assay. Data presented as mean ± SEM. *P < 0.05; **P < 0.01; ***P < 0.001.

Cuproptosis, a newly identified form of RCD, may be able to generate immunogenic signals, such as DAMPs, which could rouse the immune system. Therefore, the in vitro ICD effect induced by ES-Cu-MOF nano-regulators was evaluated in fibrosarcoma. Following treatment with different formations, Western blotting was employed to measure the extracellular CARL and HMGB1 levels in cell supernatant. The results indicated that the CARL and HMGB1 levels in the ES-Cu-MOF group were significantly higher than those in the PBS, ES, and Cu-MOF groups (Fig. 4A). Additionally, CLSM showed that the outer layer of ES-Cu-MOF- and ES-Cu-treated groups had CARL exposed, thus verifying the ICD in tumor cells induced by ES-Cu-MOF (Fig. 4B). The ATP assay kit was used to measure the extracellular levels of ATP in MCA205 cells, as a supplementary marker for ICD. The results showed that the ATP levels in the ES-Cu-MOF group were significantly higher than those in the PBS, ES, and Cu-MOF groups (Fig. 4C), similar to the positive control ES-Cu. These results demonstrated that ES-Cu-MOF nano-regulators could effectively induce ICD in MCA205 cells.

To evaluate the immunogenic properties of ES-Cu-MOF nano-regulators, the surface expression of costimulatory molecules (CD80 and CD86) on BMDCs after co-culture was assessed, as these molecules are associated with DC maturation. As depicted in Fig. 4D, a BMDCs–tumor cells coculture assay was employed to assess the influence of ES-Cu-MOF nano-regulators on DC maturation. MCA205 cells were first exposed to the nano-regulators for 6 h, after which immature BMDCs were added to the post-treated MCA205 cells for 24 h. Flow cytometry was then used to examine the maturation status of the DCs. The results showed that the expression of CD80 and CD86 on BMDCs after co-culture with ES-Cu-MOF-treated MCA205 cells was significantly higher than that of the other groups (Fig. 4E). The ES-Cu-MOF-treated group induced 55.4% mature DC, which was 1.72-fold and 2.29-fold higher than the ES-treated group (32.7%) and Cu-MOF-treated group (24.0%), respectively (Fig. 4F). To gain a more comprehensive understanding of the activation of T cells, the T cells from the spleen lymphocytes of C57BL/6 mice were collected and labeled with CFSE. Subsequently, they were incubated with BMDCs for 48 h at 37 °C, with the BMDCs being pre-treated as mentioned previously (Fig. 4G). The flow cytometry analysis revealed that the proliferation of CD8^+^ T cells was significantly augmented with the ES-Cu-MOF treatment (Fig. 4H). Compared to the PBS control, the ES-Cu-MOF group exhibited a 3.2-fold increase in proliferation, while the ES group showed a 1.94-fold increase (Fig. 4I). These results confirm that ES-Cu-MOF-induced ICD of fibrosarcoma can effectively stimulate DC maturation and induce the proliferation of CD8^+^ T cells in vitro. Overall, ES-Cu-MOF nano-regulators have the capability to significantly boost antitumor responses by reliably activating DCs.

### Anti-tumor therapy of ES-Cu-MOF nano-regulators in vivo

**Fig. 5.**
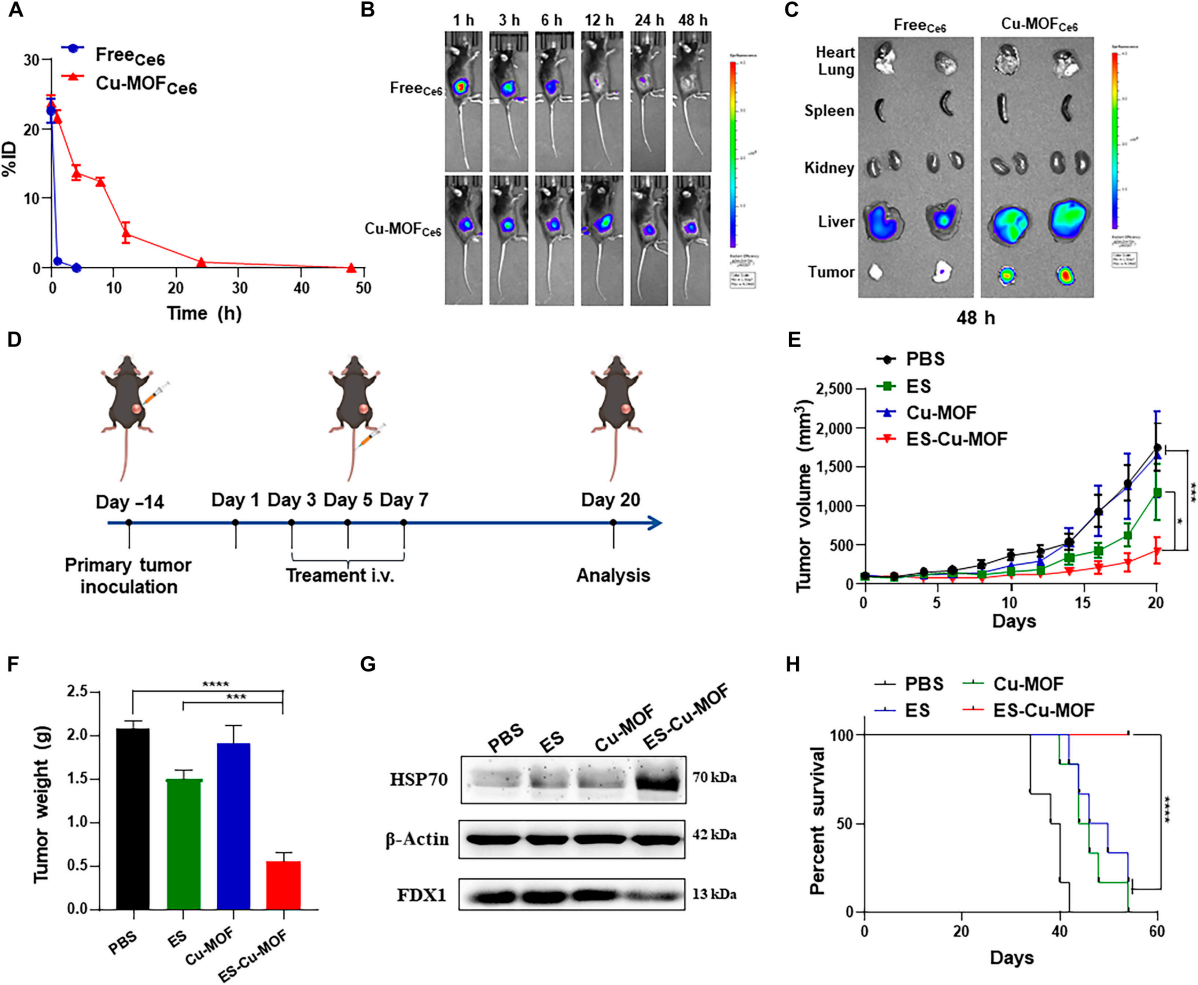
In vivo targeted delivery, tumor accumulation, and anti-tumor performance of the ES-Cu-MOF nano-regulator. (A) The pharmacokinetics of FreeCe6 and Cu-MOF_Ce6_. (B) Biodistribution after intravenous injection of FreeCe6 and Cu-MOFCe6 at different time points. (C) Tumor accumulation 48 h after the injection. (D) Schematic illustration of the treatment strategy. (E) The tumor growth curves in MCA205 tumor-bearing mice following various treatments. (F) The weight of the tumor mass was determined at the end of treatment. (G) The Western blot analysis of FDX1 and HSP70 expression in tumor tissues following treatments. (H) Survival curves in MCA205 tumor-bearing mice following various treatments. Data presented as mean ± SEM. *P < 0.05;****P < 0.001; ****P < 0.0001.

The success of the in vitro experiments prompted further investigation into the ES-Cu-MOF nano-regulators as a potential therapeutic treatment in vivo. First of all, an adequate accumulation of agents in the tumor was essential to initiate an effective cuproptosis and ICD-induced antitumor immunity. To explore the bio-distribution and pharmacokinetics of Cu-MOF nanoplatforms in vivo, Ce6 was used as a probe in the experiment, denoted as Cu-MOF_Ce6_. An investigation into the biodistribution of Cu-MOF_Ce6_, administered intravenously at a dose of 2 mg/kg was conducted in mice with tumors. In order to assess the pharmacokinetics of Cu-MOF_Ce6_, it was administered intravenously to tumor-bearing mice. The results presented in Fig. 5A and Fig. S2A demonstrated that Free Ce6 had vanished from the blood after 1 h of injection, while the fluorescence of Cu-MOF_Ce6_ was still present after 24 h. The fluorescence signal of mice injected with Cu-MOF_Ce6_ was monitored using an IVIS. Data showed that the fluorescence intensity at the tumor location augmented gradually until it reached its highest point at 12 h. On the other hand, the free Ce6 group showed a decrease in fluorescence intensity in the tumor after 6 h of injection. A remarkable fluorescence signal at the tumor site was observed even 48 h after the injection, demonstrating a high accumulation and prolonged retention of Cu-MOF_Ce6_ at the tumor site (Fig. 5B and Fig. S2B). After 48 h, the mice were sacrificed and the biodistribution of Cu-MOF_Ce6_ was evaluated ex vivo, with the highest fluorescence signal intensity observed in the tumors, surpassing that of the hearts, livers, spleens, kidneys, and lungs; thus, it was evident that Cu-MOF_Ce6_ had effectively targeted the tumors (Fig. 5C). This result suggested that MOF_Ce6_ has a prolonged circulation time, thus indicating its effectiveness in tumor enrichment.

In order to further demonstrate the therapeutic efficacy of ES-Cu-MOF nano-regulators, an in vivo therapeutic experiment was conducted in tumor-bearing mice. As illustrated in Fig. 5D, MCA205 cells were implanted into the left of female C57BL/6 mice subcutaneously and the tumor-bearing mice were randomly allocated to treatment groups. For an in vivo antitumor study, treatment formulations were systemically administered every other day and monitored bi-daily for 20 days following injection. The treatment groups with free ES slightly delayed tumor growths compared to PBS and Cu-MOF groups. It was noteworthy that the ES-Cu-MOF treatment group displayed a considerable decrease in tumor growths (Fig. 5E). During the therapeutic experiment, the body weight of the mice was monitored and no noteworthy decrease was seen after the varied treatments (Fig. S3A). Moreover, the photos of dissected tumor tissues from different treatment groups (Fig. S3B) and the mass of tumor tissues (Fig. 5F) demonstrated a similar trend in the tumor volume. No significant changes were observed in the histology of the major organs, apart from renal injury caused by free ES (Fig. S3C). The examination of the blood biochemical and physiological parameters of mice administered with different drugs indicated that mice treated with ES-Cu-MOF did not display a considerable variation in their alanine aminotransferase (ALT) and aspartate aminotransferase levels in comparison to those treated with PBS, while ALT in mice treated with free ES was increased (Fig. S3D and E).

FDX1 was identified as the pivotal gene involved in cuproptosis. First of all, to investigate the correlation of FDX1 with the immune microenvironment in fibrosarcoma, we analyzed the expression of FDX1 in normal and tumor tissue. The results showed that FDX1 was significantly down-regulated in tumor tissue compared to normal tissue (Fig. S4A). An investigation into the relationship between disease-free survival and FDX1 expression in sarcoma patients showed that those with low FDX1 expression experienced a longer disease-free survival than those with high FDX1 expression, as illustrated in Fig. S4B. To conclude the cuproptosis, the expression of FDX1 in tumor tissues from the different groups was assessed through Western blotting. Results from the ES-Cu-MOF treatment group showed a significant decrease in FDX1, as well as an elevated level of HSP70 (Fig. 5G). To further confirm the therapeutic efficacy of ES-Cu-MOF, a tumor-bearing mice survival experiment was conducted. The results revealed that the mice treated with ES-Cu-MOF had a significantly increased survival rate compared to other formulations, surviving for more than 60 days (Fig. 5H). Together, these data confirmed that the ES-Cu-MOF nano-regulator cancer therapy was able to postpone tumor growth and initiate cuproptosis of tumor cells, while also displaying low toxicity and satisfactory safety.

### Anticancer immune response of ES-Cu-MOF nano-regulators in vivo

**Fig. 6.**
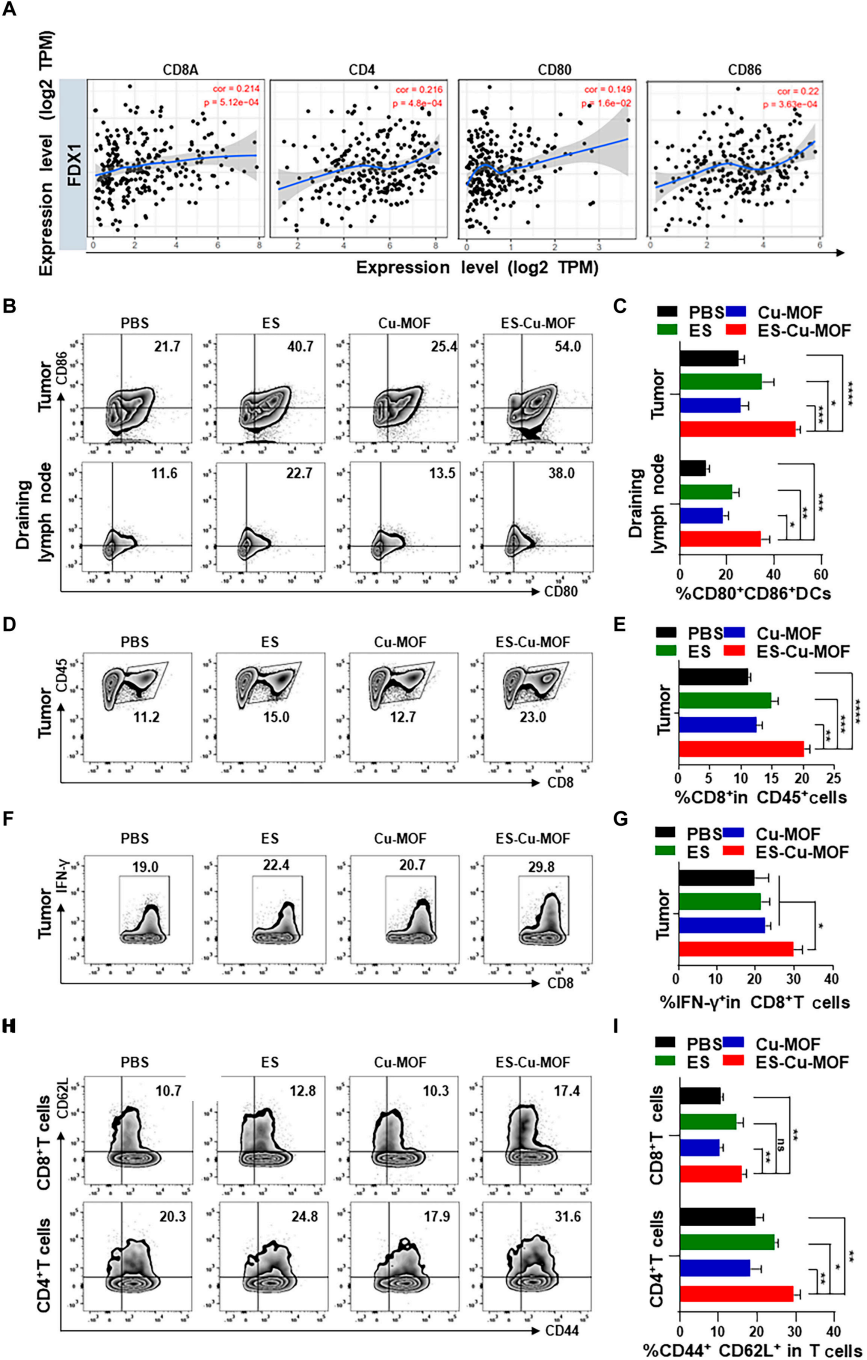
In vivo immune responses after ES-Cu-MOF nano-regulator therapy. (A) Positive correlation of FDX1 expression with immune infiltration level (CD8a, CD4, CD80, and CD86) in fibrosarcoma. (B and C) Representative flow cytometric plots and statistical percentages of mature DCs (CD80^+^CD86^+^) in tumor and draining lymph nodes after treatment. (D and E) Representative flow cytometric plots and statistical percentages of CD8^+^ T cells in tumor after treatment. (F and G) Representative flow cytometric plots and statistical percentages of IFN-γ in CD8^+^ T cells from tumor after treatment. (H and I) Representative flow cytometric plots and statistical percentages central memory T cells (CD44^+^CD62L^+^) among CD8^+^ T cell subsets and CD4^+^ T cell subsets in tumor after treatment. Data presented as mean ± SEM. *P < 0.05; **P < 0.01; ***P < 0.001; ****P < 0.0001.

ES-Cu-MOF nano-regulators have been demonstrated to be capable of inducing cuproptosis and effectively promoting ICD in vitro. To validate the immunological effects in vivo, the relationship between FDX1 expression and the infiltration of immune cells, as well as the expression of immune factors, has also been studied. As shown in Fig. 6A, FDX1 expression was found to be positively correlated with the percentage of CD8^+^ T cells and CD4^+^ T cells in fibrosarcoma. The results also revealed that FDX1 was significantly positively correlated with expression of costimulatory molecules CD80 and CD86 in fibrosarcoma. These demonstrated that the FDX1 protein levels were associated with the immune microenvironment in fibrosarcoma. ES-Cu-MOF nano-regulators may likely result in cuproptosis and concurrently evoke an anti-tumor immune response in vivo. The ICD effect of ES-Cu-MOF nano-regulators was the key to increase the maturation of DCs and active immune responses; CARL as ICD indicator was first evaluated by immunofluorescence staining. On day 2 after injection, the ES-Cu-MOF treatment groups displayed a substantial increase in CARL expression in tumor tissues (Fig. S5A and B) compared to the other groups. Additionally, the ATP assay kit revealed a notable rise in extracellular ATP levels in the tumor tissue, a sign of ICD, due to ES-Cu-MOF nano-regulator therapy (Fig. S5C).

To gain a deeper insight into the process of immune reactions after various treatments, flow cytometry was initially employed to analyze the activation of DCs in tumors and the lymph nodes that drain them, following treatment. Compared to the PBS group (21.7%), the ES-Cu-MOF treatment group had a 1.95-fold increase of 54.0%, the highest amount of mature DC. The free ES-treatment group, meanwhile, elicited a fraction of mature DC (40.7%). ES-Cu-MOF- treatment resulted in a significantly higher proportion of mature DC (38.0%) in TDLNs compared to any other group. These findings are consistent with the DC activation observed in tumors (Fig. 6B and C). DC activation demonstrated that ES-Cu-MOF treatment could potentially enhance antitumor T cell immunity. This was further validated by measuring the proportion of cytotoxic CD8^+^ T lymphocytes (CTLs) in tumors using flow cytometric analysis (Fig. S6). The ES-Cu-MOF treatment group demonstrated the most significant infiltration of CD8^+^ T lymphocytes in tumor tissues (23.0%), which is in line with its role in tumor suppression (Fig. 6D and E). Moreover, the percentage of IFN-γ^+^CD8^+^ T lymphocytes was higher than that of the other control groups (Fig. 6F and G). After 20 days of treatment, the percentage of central memory T cells (CD44^+^CD62L^+^) among the CD8^+^ T subset and the CD4^+^ T subset in the tumor from mice was significantly higher than that of the PBS group, with 17.4% and 31.6%, respectively (Fig. 6H and I). The immunofluorescence assay revealed that ES-Cu-MOF treatment induced the infiltration of CD8^+^T cells into the tumors, which was significantly more than the other groups (Fig. S5D). These results consequently demonstrated the successful establishment of antitumor T cell immunity.

### Systemic antitumor immunity of ES-Cu-MOF nano-regulators in vivo

**Fig. 7.**
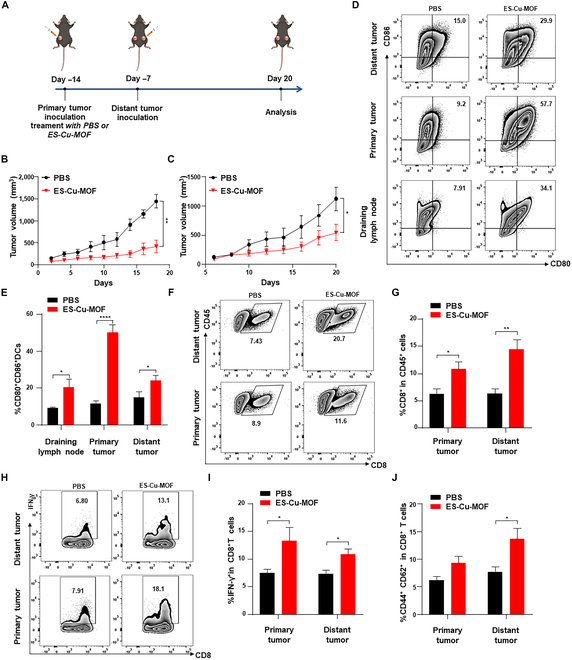
In vivo systemic antitumor immunity after ES-Cu-MOF nano-regulator treatment. (A) Schematic illustration of the systemic antitumor immunity strategy. (B and C) The growth curves of primary tumors and distant tumors after PBS or ES-Cu-MOF treatment. (D and E) Representative flow cytometric plots and statistical percentages of mature DCs (CD80^+^CD86^+^) in primary tumors, distant tumors, and draining lymph nodes after treatment. (F and G) Representative flow cytometric plots and statistical percentages of CD8^+^ T cells in primary tumors and distant tumors after treatment. (H and I) Representative flow cytometric plots and statistical percentages of IFN-γ in CD8^+^ T cells from primary tumors and distant tumors after treatment. (J) Statistical percentages of central memory T cells (CD44^+^CD62L^+^) among CD8^+^ T cell subsets and CD4^+^ T cell subsets in primary tumors and distant tumors after treatment. Data presented as mean ± SEM. *P < 0.05; **P < 0.01.

Finally, the mechanism of systemic antitumor immunity induced by ES-Cu-MOF nano-regulators was investigated. As shown in Fig. 7A, a bilateral tumor system was employed, wherein MCA205 cells were implanted into the left side of female C57BL/6 mice subcutaneously after treatment with PBS or ES-Cu-MOF as primary tumor, and the same number of tumor cells was implanted into the right to mimic distant tumor after 7 days. Tumor growth of primary and distant tumors was monitored every 2 days for 20 days. The results showed that ES-Cu-MOF treatment significantly inhibited the growth of primary and distant tumors (Fig. 7B and C). This indicates that ES-Cu-MOF treatment could induce systemic antitumor immunity. To further investigate the mechanism of systemic antitumor immunity, the activation of DCs and the infiltration of CD8^+^ T lymphocytes in tumor tissues were measured by flow cytometry. As shown in Fig. 7D and E, ES-Cu-MOF treatment significantly increased the proportion of mature DC in primary and distant tumors and draining lymph node of distant tumors. The infiltration of CD8^+^ T lymphocytes and IFN-γ^+^CD8^+^ T lymphocytes in primary and distant tumors was also significantly increased (Fig. 7F to I). Additionally, the number of central memory T cells (CD44^+^CD62L^+^) among the CD8^+^ T subset in distant tumors was greater than the levels observed in the PBS (Fig. 7J). Our findings indicated that ES-Cu-MOF treatment could stimulate systemic antitumor immunity by activating DCs, resulting in the infiltration of CD8^+^ T lymphocytes and the formation of immune memory. The above results served as evidence of the successful establishment of systemic antitumor immunity.

## Discussion

RCD held promise for effectively inducing ICD and stimulating systemic immune responses to enhance anti-tumor effects [4,5,16–18]. It was crucial to explore novel strategies to facilitate the release of immunogenic molecules, particularly in tumors with low immunogenicity. Cuproptosis, a copper-dependent form of RCD, was recognized for its potential to boost the immunogenicity of tumors and improve therapeutic outcomes [30]. In response to this potential, we developed metal ion nano-regulators aimed at initiating cuproptosis and bolstering anti-tumor immunity.

Recent advancements had seen the integration of MOFs into clinical scenarios, particularly for drug delivery [38–41]. MOFs were particularly effective in spontaneously releasing metal ions such as Fe, Cu, and Mn within tumor cells, positioning them as excellent candidates for metal ion delivery systems [42]. Leveraging this property, we engineered a Cu-MOF infused with ionophores like ES, creating an innovative method for inducing cuproptosis and subsequent ICD in tumors with inherently low immunogenicity. Compared to other modalities, our Cu-MOF nano-regulator embedded with ES (ES-Cu-MOF) successfully released Cu^2+^ and ES in response to cellular conditions, fostering mitochondrial ROS generation and promoting cuproptosis in cancer cells. This nano-regulator effectively induced a cuproptosis burst dependent on FDX1 and triggered ICD, enhancing immune system engagement.

ES-Cu-MOF significantly escalated mitochondrial oxidative stress in cellular models, facilitating the release of DAMPs such as CARL and HMGB1 from dying tumor cells. This process activated DCs, prompting robust anti-tumor immune responses. Evidence suggested that integrating copper-based cuproptosis induction with immunotherapy that stimulated cuproptosis led to superior anti-tumor outcomes. Intravenous administration of ES-Cu-MOF nanoparticles concentrated these agents at tumor sites, effectively inhibiting fibrosarcoma growth and enhancing cytotoxic CD8^+^ T cell infiltration, thereby improving immune responses against the tumor. Both primary and distant tumor growth control, coupled with significant survival rate improvements, highlighted the efficacy of this systemic antitumor immunity, suggesting its broad application potential across various tumor stages.

Although this study has achieved good therapeutic results and has shown promising application prospects, there are still areas for further improvement. Future research should focus on optimizing the formulation for enhanced stability and specificity, reducing potential off-target effects, and further exploring the underlying molecular mechanisms. Additionally, clinical translation of this nanotechnology demands a comprehensive evaluation of its safety profile across diverse patient populations to ensure its efficacy and minimization of adverse effects. This study lays a promising foundation for harnessing the power of metal-based nanotechnology in cancer treatment, marrying the traditional benefits of chemotherapy with the novel insights of immunotherapy.

Prompting tumor cells to undergo RCD had potential to effectively induce ICD and stimulate systemic anti-tumor immune responses, and subsequently improve antitumor effects. It was essential to explore different approaches to promote the release of immunogenic molecules in low immunogenic tumors. In this study, a pioneering approach to cancer immunotherapy was developed by leveraging a Cu-MOF nanoplatform embedded with ES, crafting a novel pathway to provoke cuproptosis and subsequent ICD in low immunogenic tumors. The incorporation of ES into Cu-MOFs nanoplatform has demonstrated potential to induce cuproptosis burst in an FDX1-dependent manner and stimulate tumor cell death in an immunogenic manner, thus improving the survival rate of tumor-bearing mice. This nano-regulator was able to release Cu^2+^ and ES in response to the intracellular environment, resulting in increased mitochondrial ROS generation and cuproptosis of tumor cells. Additionally, it was able to trigger ICD to activate the immune system. Studies have demonstrated the superior antitumor effects of combining copper-based cuproptosis induction and cuproptosis-stimulated immunotherapy, with intravenous injection of the ES-Cu-MOF nanoparticles enriching the tumor site and inhibiting the growth of fibrosarcoma, leading to enhanced cytotoxic CD8^+^ T infiltration and improved antitumor immune responses.

In summary, we developed a metal-based nanotechnology that not only triggers cuproptosis but also elicits a powerful ICD for systemic anti-tumor immune response. The established Cu-MOF nanoplatform with biocompatibility potentially provided a novel approach for anti-tumor immunotherapy.

## Data Availability

Data will be made available on request.
