# Supplementary material for: Copper(II)-Based Nano-Regulator Correlates Cuproptosis Burst and Sequential Immunogenic Cell Death for Synergistic Cancer Immunotherapy
Source: Biomater Res. 2024 Jun 27;28:0039. doi: 10.34133/bmr.0039 (PMC11208873; doi:10.34133/bmr.0039)
Supplement: Supplementary 1 — Table S1 Figs. S1 to S6 [file bmr.0039.f1.docx]

**Copper(II)-Based Metal-Organic Frameworks Nanoplatforms Nano-regulator Correlates Enhance Cuproptosis burst and Sequential Immunogenic Cell Death for Synergistic Cancer Immunotherapy**

1. **Supporting Figures**

**Table S1 The percentage of different elements detected in Cu-MOF and ES-Cu-MOF**

**
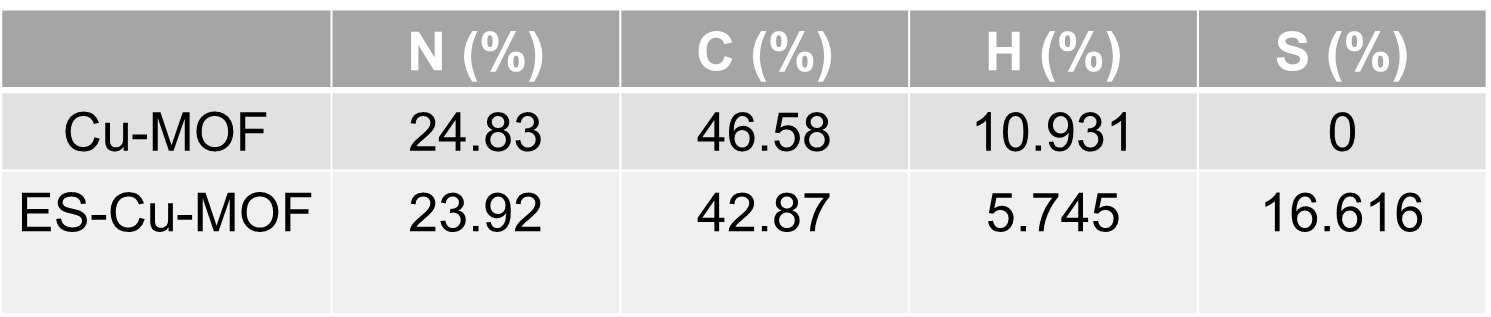
**

**Figure S1. *In vitro* cuproptosis induction of ES-Cu-MOF nano-regulator**. (A) Mean ﬂuorescence intensity (MFI) of Cu2+ in MCA205 cells after treatment. Flow cytometric plots illustrating the cellular uptake of Cu-MOF_Ce6_ at different time points. (B) Cell viability of HT1080 cells treated with the indicated concentrations of elesclomol for 6 hours. Quantitative statistical results of HSP70 (C) and FDX1 (D). (E) Immunoblot of FDX1 and β-actin from extracts of HT1080 cells with deletion of FDX1. (F) Cell viability of HT1080 cell and FDX1 KO HT1080 cells treated with the indicated concentrations of elesclomol for 6 hours. (G) Intracellular ROS of MCA205 cells after treatment. Nuclei were stained by DAPI (blue), MCA205 cells were loaded with ROS-reactive dye H_2_DCFDA (red), Scar bar = 50 μm. Data presented as mean ± SEM.


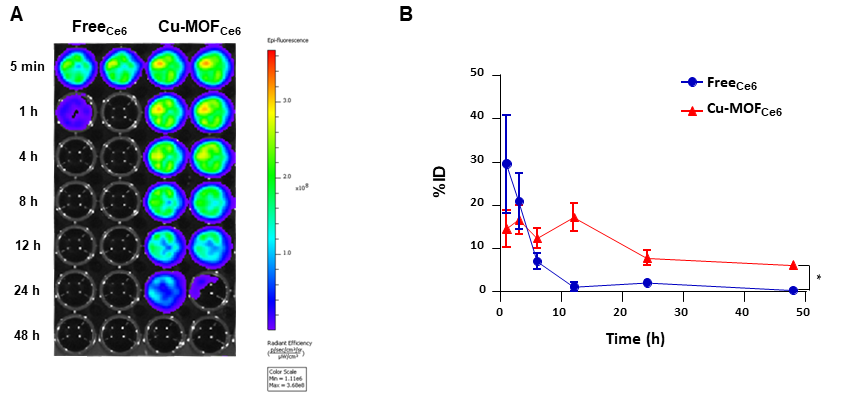


**Figure S2. *In vitro* pharmacokinetics and tumor accumulation of ES-Cu-MOF nano-regulator.** (A) Immunofluorescence imaging of Cu-MOF_Ce6_ from the blood at different time points. (B) Tumor accumulation at different time points. Data presented as mean ± SEM.

**Figure S3. *In vivo* anti-tumor performance and systemic toxicity of ES-Cu-MOF nano-regulator.** (A) Body weights of mice during the treatment. (B) Image of the MCA205 tumors at the end of treatment. (C) Representative H&E staining of major organs (heart, liver, spleen, lung, kidney and tumor) of mice after different treatments. (D) ALT of the serum of mice with various treatments. (E) AST of the serum of mice with various treatments. Data presented as mean ± SEM.

**
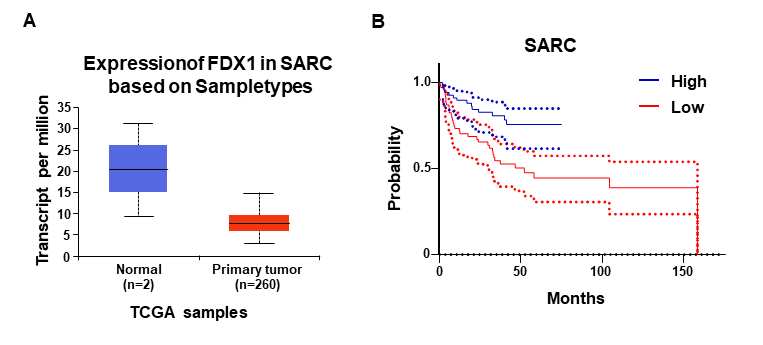
**

**Figure S4. Correlation of Cuproptosis-Related Gene FDX1 with Fibrosarcoma.** (A) The expression of FDX1 in normal and tumor tissue. (B) Correlation of disease-free survival with FDX1 expression in fibrosarcoma patients using GEPIA.


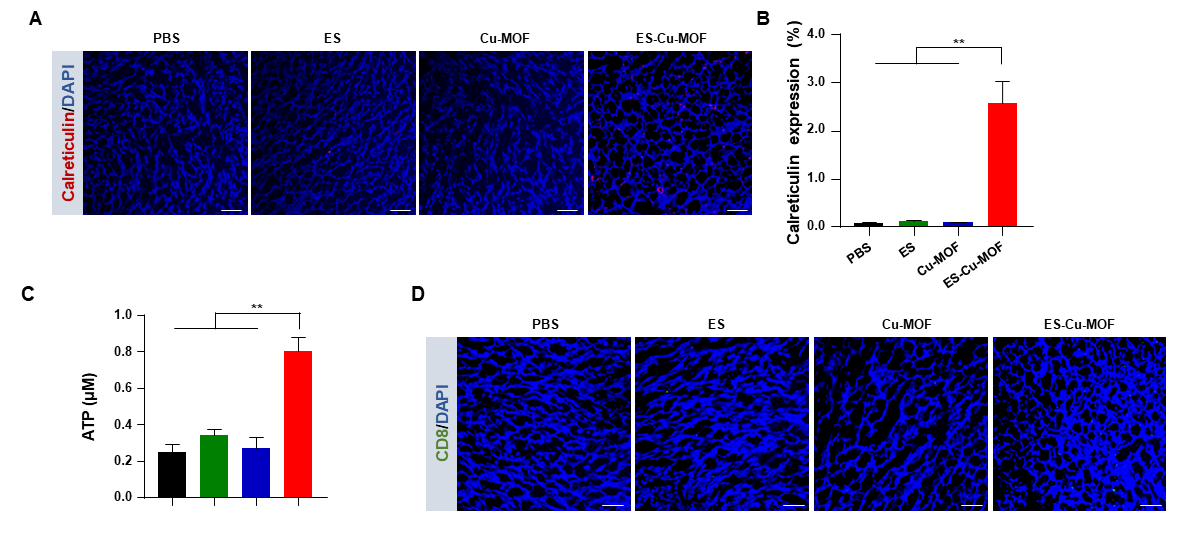


**Figure S5. *In vivo* ICD induction and immune responses of ES-Cu-MOF nano-regulator**. (A) Immunofluorescence staining of CARL (red) of tumor tissues from mice following various treatments. (B) Quantitative analysis of the expression level CRT in images from (A) (n = 3). (C) The release of ATP in MCA205 tumors after different treatments by ATP assay kit. (D) Immunofluorescence staining images of CD8^+^ T cell in tumor sections from mice following various treatments. Data presented as mean ± SEM.


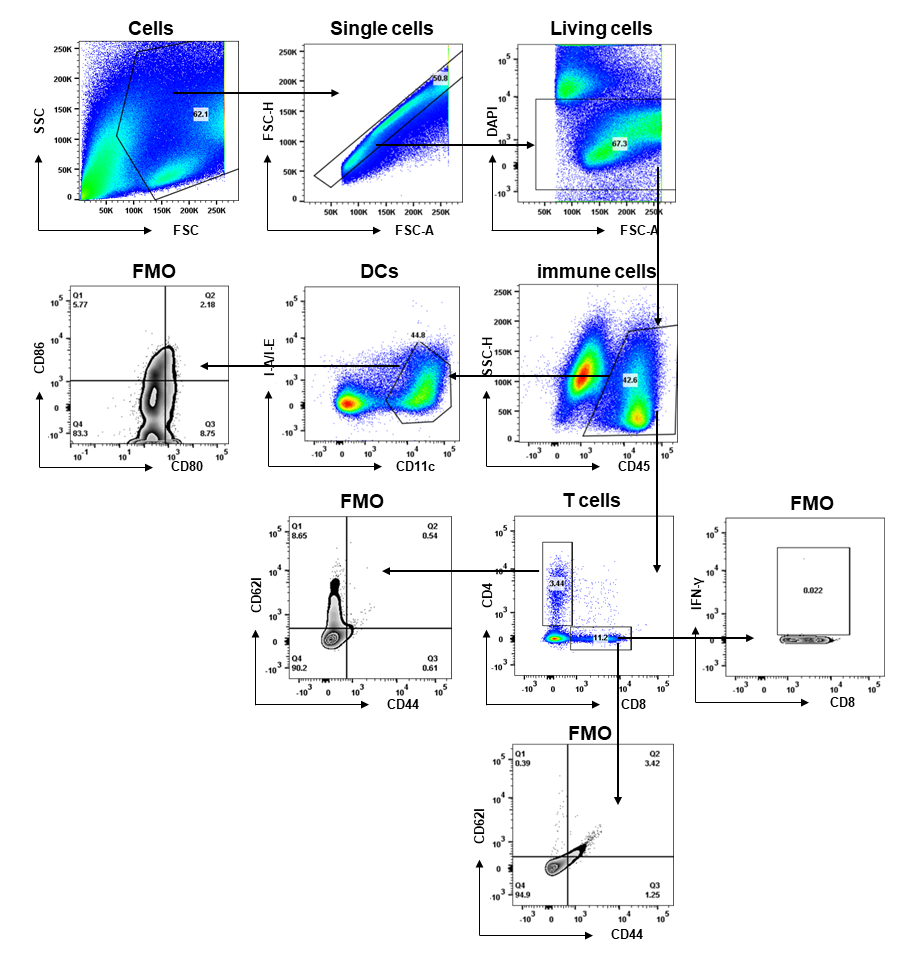


**Figure S6.** Flow cytometry gating strategy to identify DCs, CD4^+^/CD8^+^ T cell subsets and central memory T cells among CD4^+^/CD8^+^ T cell subsets and related FMO controls in tumor tissues.
